# Correlation Between Physical Activity and Psychological Problems in Secondary School Students in Spain

**DOI:** 10.3390/sports13100362

**Published:** 2025-10-11

**Authors:** Pablo Pueyo Gutiérrez-Rivas, Demetrio Lozano, Alberto Roso-Moliner, Rafael Albalad-Aiguabella, Oscar Villanueva-Guerrero, Elena Mainer-Pardos

**Affiliations:** Health Sciences Faculty, Universidad San Jorge, Autovía A23 km 299, Villanueva de Gállego, 50830 Zaragoza, Spain; ppueyogr@gmail.com (P.P.G.-R.); dlozano@usj.es (D.L.); aroso@usj.es (A.R.-M.); ralbalad@usj.es (R.A.-A.); ovillanueva@usj.es (O.V.-G.)

**Keywords:** physical activity, mental health, adolescence, secondary education, DASS-21, gender, educational stage, individual sports, team sports

## Abstract

Physical activity (PA) has been identified as a protective factor for adolescent mental health. This study analysed the association between PA and levels of anxiety, depression, and stress among adolescents, considering gender, educational stage, and type of sport. A cross-sectional design was conducted with 106 Spanish secondary school students aged 12–16 years. Data were collected through a self-reported questionnaire on PA participation and the validated DASS-21 scale. Descriptive statistics, chi-square (χ^2^) tests, and adjusted residual analyses were performed. The results showed a significant negative association between PA and anxiety (χ^2^ = 303.34, *p* < 0.01), stress (χ^2^ = 310.64, *p* < 0.01), and depression (χ^2^ = 324.32, *p* < 0.01). Non-athletes presented higher levels of psychological problems compared with active peers, while girls and older students showed greater vulnerability. Adolescents involved in team sports exhibited lower anxiety and stress than those participating in individual sports. In conclusion, higher participation in physical activity, particularly team-based disciplines, is associated with better mental health in adolescents. These findings reinforce the importance of integrating regular physical activity into school contexts to support psychological well-being during adolescence.

## 1. Introduction

In recent decades, adolescent mental health has become a global concern, with a notable increase in disorders such as anxiety, depression, stress, low self-esteem, and disruptive behaviors [1,2,3,4]. These problems are strongly influenced by academic pressure, family instability, social uncertainty, excessive use of digital technologies, and constant exposure to social networks, which have been linked to increased risks of anxiety, sleep disturbances, and low self-esteem, while also shaping peer relationships and social comparison among adolescents [4]. The COVID-19 pandemic further aggravated this scenario, as social isolation and reduced opportunities for physical activity intensified emotional problems such as anxiety, depression, and stress among young people [1,5,6].

Adolescence, typically between ages 12 and 18, represents a critical developmental stage marked by profound physical, cognitive, and social transformations [7,8]. During this period, individuals consolidate their personal identity, autonomy, and values and establish habits that will influence their adult life [9]. However, this stage is also associated with heightened vulnerability to psychological imbalances, as adolescents face internal and external challenges that may compromise their well-being [3,10].

Educational institutions play a fundamental role in adolescent development, not only by providing academic instruction but also by fostering socio-emotional competencies. Schools are privileged spaces for the early detection of psychological problems and the implementation of preventive interventions [11]. Despite this, the promotion of well-being is still often secondary to academic performance. Physical Education and extracurricular sports could serve as key tools for enhancing emotional health, but they are frequently undervalued and underutilized [12,13]. Concrete strategies may include integrating structured physical activity programs into the school day, expanding extracurricular sport opportunities, and fostering collaboration with community sport organizations to ensure sustained participation. Moreover, the lack of coordination among teachers, counselors, and health professionals hampers the development of integrated health promotion programs.

Physical activity has been consistently recognized as a major protective factor for adolescent mental health [12]. Beyond structured exercise, any form of movement that increases energy expenditure has been linked to physical and psychological benefits. Regular participation in physical activity improves mood, reduces the symptoms of depression and anxiety, enhances self-esteem, and fosters emotional regulation and general well-being [1,14,15,16,17]. However, most adolescents fail to meet the international guidelines. According to the World Health Organization, approximately 80% of adolescents worldwide do not meet the recommendation of engaging in at least 60 min of moderate to vigorous physical activity per day, and in Spain, more than 60% of adolescents fail to achieve these minimum levels [15,18].

Sedentary behavior has become a pressing public health issue among adolescents [19]. Increased screen time and digital entertainment are not only associated with physical problems such as obesity but also with psychological issues, including social withdrawal, impulsivity, low self-esteem, and higher levels of stress [20,21]. During the COVID-19 pandemic, these risks intensified, but adolescents who maintained physical activity levels were associated with better mental health outcomes, highlighting the relationship between exercise and psychological well-being in times of adversity [6,22,23]. The PASOS project in Spain has revealed concerning rates of inactivity and sedentarism, along with high levels of anxiety, sleep disturbances, and body dissatisfaction among adolescents, further emphasizing the urgency of effective interventions [24].

The relationship between physical activity and mental health is complex and mediated by multiple variables. Gender differences, activity type, exercise intensity, and motivational orientation strongly influence the psychological outcomes of physical activity [16,25]. Intrinsic motivations such as enjoyment, personal challenge, and perceived competence have been shown to yield greater emotional benefits than extrinsic drivers such as social approval or body image concerns [16]. Moreover, school climate plays a decisive role: teacher support, peer relationships, and the presence of a positive and inclusive environment buffer stress and enhance the emotional benefits of physical activity [26,27]. In addition, the type of sport practiced introduces further differences: individual sports have been associated with improvements in executive functions, self-regulation, and decision-making skills [28,29], whereas team sports foster cognitive flexibility, creativity, social interaction, and teamwork, all of which are strongly linked to psychological well-being [30,31]. Conversely, competitive sport may either promote or hinder mental health, depending on coping strategies and the availability of social support [32].

Biological and neuropsychological mechanisms help explain these benefits. Exercise induces the release of serotonin, dopamine, and norepinephrine, which are neurotransmitters directly involved in mood regulation, attention, and motivation, while reducing the cortisol levels associated with chronic stress. It also stimulates neuroplasticity in regions such as the hippocampus and prefrontal cortex, which are crucial for executive functioning, decision-making, and emotional regulation [33,34,35]. These adaptations translate into better academic performance, resilience, and emotional stability [36,37,38]. Furthermore, recent studies indicate that extracurricular physical activity directly improves school performance through cognitive and social factors, reinforcing its fundamental role in education [39]. This evidence underscores the importance of integrating physical activity into the school curriculum as a pillar of holistic education that goes beyond physical health to also promote cognitive and emotional development.

Taken together, the scientific evidence highlights the role of physical activity as a multidimensional strategy to improve adolescent well-being, with schools positioned as key environments to address sedentary behavior and psychological problems. In this context, the present study aims to: (i) examine the correlation between physical activity levels and psychological problems (anxiety, depression, and stress) in adolescents from a Spanish secondary school; (ii) analyze differences in psychological problems according to gender; (iii) compare psychological problems across educational stages (1st–2nd vs. 3rd–4th grades of compulsory secondary education); and (iv) evaluate psychological problems between non-athletes and athletes, distinguishing between team and individual sports. The working hypothesis is that students who engage in sports are less likely to present with psychological problems, but among them, those practicing individual sports are at higher risk than those participating in team sports, and female students are expected to show greater psychological difficulties than males.

## 2. Materials and Methods

### 2.1. Participants

The study sample consisted of 106 students enrolled in a single public secondary education center in Spain, aged between 12 and 16 years. All students within this age range were invited to participate, and only those who returned signed parental consent forms were included. The distribution was balanced by sex, with 57 students identifying as male and 49 as female. Regarding academic grade, the sample comprised 24 students from the 1st year of compulsory secondary education (CSE), 26 from the 2nd year, 21 from the 3rd year, and 36 from the 4th year. No a priori sample size calculation was performed; the final sample (N) corresponded to the total number of students who provided consent, reflecting the entire accessible population of the school. The descriptive characteristics of the participants are presented in Table 1.

All participants and their legal guardians were informed about the purpose and procedures of the study and provided written informed consent before participation. The study protocol was approved by the Ethics Committee of Universidad San Jorge (approval code: 93-2-24/25).

### 2.2. Study Design

A cross-sectional study was conducted. This paradigm assumes that reality is objective, measurable, and can be examined using descriptive and inferential analyses.

Data collection was carried out using a structured questionnaire developed in Microsoft Forms. The instrument was divided into two sections. The first section of the questionnaire collected demographic data (sex and academic grade) and physical activity habits. Specifically, it included items on participation in physical activity outside school hours, type of sport practiced (individual, team, or no sport), and involvement in high-performance training (>15 h/week). Importantly, no personally sensitive information was requested. The second section consisted of the Depression, Anxiety, and Stress Scale–21 (DASS-21), a validated and widely applied instrument in adolescent populations [40]. This scale assesses stress, anxiety, and depression through 21 items rated on a four-point Likert scale ranging from 0 (“Did not apply to me at all”) to 3 (“Applied to me very much or most of the time”). Each of the three subscales comprises seven items, and scores are obtained by summing the responses within each dimension. Higher scores indicate greater severity of symptoms in the respective constructs.

### 2.3. Procedures

The school administration distributed a detailed report to parents and legal guardians via the school’s communication platform. This report included information about the study objectives, the rationale, and a copy of the questionnaire. Participation required signed informed consent from parents or legal guardians, which was returned to the principal investigator. Only students with prior parental authorization were invited to complete the questionnaire. All procedures were carried out in collaboration with the school principal during the regular school hours.

Parents and guardians were explicitly informed that the questionnaire was anonymous, voluntary, and carried no risks or benefits associated with participation. Before administration, the students received a detailed explanation from the principal investigator regarding the aims of the study and the questionnaire’s content. They were also provided with a written Participant Information Sheet and given time to read it carefully and ask any questions before proceeding. During this time, students could ask questions directly to the investigator. It was emphasized again that participation was voluntary and that they could decline to participate, even if parental consent had been previously granted.

The questionnaire was administered during each group’s weekly tutorial session. Students completed the survey in the school’s computer laboratory using a secure link provided by the school administration. The process was supervised with the support of the school principal, ensuring confidentiality and a standardized administration procedure.

### 2.4. Data Analysis

After reviewing the nature of our variables, we decided to apply the Pearson Chi-square test, as both physical activity (PA) and psychological distress levels (DASS-21) were categorized in our analysis. This test allowed us to assess whether a significant association existed between these two categorical variables, which aligned with the objectives of our study. To further analyze the results, the statistical test ‘corrected residuals’, also known as ‘adjusted residuals’, was used. This is a powerful tool for accurately interpreting the significance of the associations detected above. This statistical test allows us to accurately interpret the significance of the relationships between categories in terms of the standardized z-score. The standardized z-score data are shown in the adjusted residual contingency tables, presenting excitatory patterns with z-values greater than 1.96 and inhibitory patterns with z-values less than −1.96. Z-score values ≥ ±1.96 or <±2.58 correspond to *p* < 0.05 (*), z-scores ≥ ±2.58 or <±3.29 correspond to *p* < 0.01 (**), and z-scores ≥ ±3.29 correspond to *p* < 0.001 (***). In other words, the higher the absolute value of the adjusted residual, the greater the relationship between the categories.

We also conducted an internal consistency analysis by calculating Cronbach’s alpha and McDonald’s omega for each subscale (depression, anxiety, and stress). Acceptable values for both alpha and omega (0.70 to 0.95) indicate adequate reliability of the scale within our sample.

All data were recorded in Microsoft Excel and subsequently analyzed using IBM SPSS Statistics software (Version 28.0, IBM SPSS Inc., Chicago, IL, USA).

## 3. Results

Table 1 displays the descriptive data of the participants. The sample consisted of 106 students (54% male, 46% female) aged between 12 and 16 years, distributed across the four years of secondary education. Participation in physical activity was higher among boys (92%) than girls (77%), and collective sports were the most frequently practiced type in both genders.

A descriptive statistics analysis and chi-square (χ^2^) values were obtained, and it was complemented with the adjusted residuals test.

The comparison by gender using the chi-square (X^2^) test showed significant differences in anxiety (X^2^ = 365.65, *p* < 0.05) and stress (X^2^ = 348.02, *p* < 0.05). However, no significant differences were found in depression (U = 442.55, *p* > 0.05) (Table 2).

The comparative analysis using the chi-square (X^2^) test across educational stages revealed statistically significant differences only in stress levels (X^2^ = 477.81, *p* < 0.05). No significant differences were observed for anxiety (X^2^ = 397.56, *p* > 0.05) or depression (X^2^ = 493.31, *p* > 0.05) (Table 3).

The comparison between the groups (athletes or non-athletes) using the chi-square (X^2^) test identified significant differences in all three psychological problems analyzed. Anxiety (X^2^ = 303.34, *p* < 0.01), stress (U = 310.64, *p* < 0.01), and depression (U = 324.32, *p* < 0.01).

Among the students who engaged in sports, the chi-square (X^2^) test revealed that those practicing individual or collective sports reported significantly higher levels of anxiety (X^2^ = 172.34, *p* < 0.05) and stress (U = 179.33, *p* < 0.05) and no significant differences were found for depression (X^2^ = 188.45, *p* > 0.05).

The comparison by gender using the adjusted residuals test showed significant differences in anxiety in females (Z = 2.23, *p* < 0.05) and stress (Z = 12.12, *p* < 0.05). However, no significant differences were found in depression (Z = 1.7, *p* > 0.05) (Table 4).

The comparative analysis using the adjusted residuals test across educational stages revealed statistically significant differences only in stress levels (Z = −2.45, *p* < 0.05), which were higher among students in the 3rd and 4th year of secondary education. No significant differences were observed for anxiety (Z = 1.45, *p* > 0.05) or depression (Z = 1.04, *p* > 0.05) (Table 5).

The comparison between groups (athletes or non-athletes) using the adjusted residuals test identified significant differences in all three psychological problems analyzed. Non-athletes reported higher levels of anxiety (Z = 3.12, *p* < 0.01), stress (Z = 2.93, *p* < 0.01), and depression (Z = 2.74, *p* < 0.01) compared with their physically active peers (Table 6).

Among the students who engaged in sports, the adjusted residuals test revealed that those practicing individual sports reported significantly higher levels of anxiety (Z = 2.23, *p* < 0.05) and stress (Z = 1.97, *p* < 0.05) compared with those participating in team sports. No significant differences were found for depression (Z = 1.12, *p* > 0.05) (Table 7).

## 4. Discussion

The aim of this study was to examine the relationship between physical activity and psychological well-being in secondary school students, focusing on anxiety, depression, and stress in relation to gender, educational stage, and type of sport practiced. The present study identified a consistent negative association between physical activity and psychological problems in adolescents, supporting the view that higher engagement in physical activity is linked to lower levels of anxiety, stress, and depression. Notably, non-athletes, females, older students, and those engaged in individual sports showed greater vulnerability to these problems, whereas participation in team sports appeared to act as a protective factor. Although subgroup differences should be interpreted cautiously due to sample size limitations, these findings reinforce the role of physical activity as a potential buffer against mental health difficulties during adolescence, a period of heightened developmental and psychosocial demands [13,41,42].

The inverse association between physical activity and the three DASS-21 values observed in this study is consistent with previous evidence emphasizing the mental health benefits of regular exercise during adolescence [13,43]. Specifically, our results demonstrated that higher levels of physical activity were significantly associated with lower levels of anxiety (X^2^ = 303.34, *p* < 0.01), stress (X^2^ = 310.64, *p* < 0.01), and depression (X^2^ = 324.32, *p* < 0.01), reinforcing the association between exercise and mental health outcomes. In line with this, the meta-analysis conducted by Chen et al. [44] confirmed that regular physical activity can produce moderate improvements in both depressive and anxiety symptoms in adolescents, and the meta-analysis conducted by Wang et al. [45] found a moderate effect of exercise on reducing depressive symptoms in adolescents. Similarly, Laurier et al. [1] highlighted that physical activity enhances self-esteem, thereby contributing to greater emotional resilience when facing stressful situations. These findings may be explained by both biological mechanisms, such as improved neuroendocrine regulation and neurotransmitter release, and psychosocial mechanisms, including enhanced self-esteem, greater opportunities for social interaction, and more effective stress coping strategies [33,34,35,46,47]. Together, the results reinforce the view that participation in sport constitutes a significant protective factor in the prevention of psychological problems during secondary education.

Gender-based comparisons revealed that girls reported significantly higher levels of anxiety and stress than boys, while no significant differences were observed in depression. These results are consistent with prior research showing that adolescent girls are more prone to experiencing anxiety and stress than boys [48,49]. This vulnerability has been attributed to multiple factors, including biological influences such as hormonal changes during puberty, as well as social and cultural pressures related to body image, academic expectations, and interpersonal relationships, which tend to affect girls more strongly during adolescence [50]. These findings highlight the need for school- and community-based public health strategies that specifically promote physical activity among female adolescents, as fostering active lifestyles is associated with lower vulnerability to anxiety and stress and supports healthier developmental trajectories. Nevertheless, given the limited subgroup sample size, these sex-based findings should be interpreted with caution and confirmed in larger studies.

Educational stage also emerged as a relevant variable, with older students (3rd and 4th year of secondary education) reporting significantly higher stress levels than their younger peers. This is consistent with the idea that academic demands, increased social pressures, and concerns about future academic or professional pathways may intensify stress during mid-to-late adolescence [51,52]. Similarly, Fernández-Sogorb et al. [26] identified an increase in stress among students in higher grades, attributable to rising academic demands, vocational decision-making pressures, and the emotional instability typical of the transition to adulthood. In line with this, Patton et al. [53] and Orben et al. [54] also reported that psychological distress tends to increase with age during adolescence, partly due to heightened academic demands, social pressures, and the progression toward adult responsibilities. These findings underscore the importance of implementing preventive strategies tailored to each stage of secondary education, when psychological vulnerability may be greater.

The comparison between athletes and non-athletes revealed that adolescents who did not engage in regular physical activity reported significantly higher levels of anxiety, depression, and stress (all *p* < 0.01). These findings emphasize the association of sport participation with mitigating anxiety, depression and stress, supporting the notion that regular physical activity serves as a buffer against mental health problems during adolescence. This aligns with previous evidence showing that physically active adolescents tend to exhibit better psychological well-being, lower prevalence of depressive symptoms, and enhanced stress regulation compared with their sedentary peers [13,41]. In particular, participation in organized sports has been associated not only with improved mental health outcomes but also with broader psychosocial benefits, including social connectedness, improved self-perception, and the development of coping strategies [43,55]. From a public health perspective, these findings emphasize the importance of developing school-based programs and extracurricular opportunities that promote sustained physical activity among adolescents.

The comparison between sport types revealed that adolescents engaged in team sports reported lower levels of anxiety and stress compared with their peers participating in individual sports, while no significant differences were observed in depression. These results are consistent with prior studies suggesting that team sports, through their social nature and cooperative demands, provide additional psychological benefits compared with individual disciplines [43,56]. Bisquert Bover et al. [16] emphasized that team-based activities foster socioemotional skills such as cooperation, empathy, and a sense of belonging, all of which act as protective factors against anxiety and stress. In contrast, Guo et al. [32] reported that individual sports may involve greater personal responsibility and heightened exposure to performance evaluation, which could partly explain the higher levels of distress observed among adolescents practicing these sports. Furthermore, Kajtna et al. [30] noted that team sports are associated with enhanced social support, a sense of belonging, and teamwork skills, which mitigate stress and anxiety. This agrees with the intermediate scores observed in the present study among students engaged in individual sports, who fell between non-athletes and team sport participants in terms of psychological well-being. These findings underscore the relevance of promoting team-based physical activity within educational settings as a practical strategy to foster mental health in adolescents, directly aligning with health promotion through sport and physical activity. However, given the relatively small subgroup sample sizes, these sport-type comparisons should be interpreted as preliminary and confirmed in larger and more diverse samples.

This study highlights the relevance of promoting physical activity and shows that different types of activities provide distinct psychosocial environments, underscoring the need for schools and policymakers to prioritize access to team-based opportunities as part of mental health promotion strategies [43,56]. These findings have important practical implications. Encouraging adolescents, particularly girls and older adolescent students, to engage in regular physical activity, especially in team sports, may serve as an effective preventive strategy associated with fewer psychological problems. Given the high prevalence of insufficient physical activity among adolescents worldwide [19], schools represent a crucial setting to foster sports participation. Interventions should not only aim to increase overall physical activity but also consider the type of sport and its psychosocial benefits, adapting strategies to the specific needs of different student groups. This study provides new evidence on the links between physical activity and psychological well-being in adolescents, highlighting that participation in team sports acts as a protective factor against anxiety, depression, and stress. These findings call for the implementation of targeted school-based interventions to enhance sports participation and promote adolescent mental health.

This study presents several limitations that should be acknowledged. First, physical activity was assessed using self-reported items rather than a validated quantitative scale, which may reduce measurement precision and increase reporting bias. Second, the categorical treatment of variables limited the statistical approach to chi-square analyses, preventing the estimation of effect sizes and confidence intervals. Third, the sample was restricted to a single secondary school, and no a priori power calculation was performed, which constrains the generalizability of the findings and reduces the statistical power of subgroup comparisons by sex, grade, and sport type. Fourth, the cross-sectional design precludes any causal interpretation of the observed associations. Finally, both physical activity and psychological outcomes were self-reported, which may introduce recall and social desirability biases.

The findings of this study highlight the relevance of promoting regular physical activity among adolescents to support mental health, particularly through team-based sports that foster social interaction and cooperation. Future school-based interventions should ensure equitable opportunities for participation, with special attention to the lower activity levels and higher psychological vulnerability observed among girls. Expanding research to different educational and socio-cultural contexts, using longitudinal and mixed-method approaches, will provide deeper insight into how sport participation contributes to emotional well-being and resilience during adolescence.

## 5. Conclusions

The findings revealed a significant negative association between physical activity and anxiety, depression, and stress in adolescents. Non-athletes reported higher psychological difficulties than physically active peers, confirming the protective role of sport participation. Girls and older students presented greater vulnerability to anxiety and stress, whereas adolescents involved in team sports showed lower stress and anxiety levels compared with those practising individual disciplines. Overall, participation in regular physical activity, particularly in team-based contexts, is associated with better psychological well-being during adolescence.

## Figures and Tables

**Table 1:** Reliability Estimates with Interpretation.

Subscale	Cronbach’s α	95% CI (α)	McDonald’s ω	95% CI (ω)	Interpretation
Anxiety	0.90	0.86–0.94	0.85	0.82–0.91	High reliability
Stress	0.89	0.85–0.93	0.82	0.79–0.88	High reliability
Depression	0.90	0.87–0.93	0.84	0.80–0.89	High reliability
Total	0.897		0.837		Overall good reliability

**Table 2:** Descriptive characteristics of participants by gender, academic year, physical activity participation, and type of sport (expressed in absolute numbers and percentages).

Gender			Academic Year			Physical Activity			Sport Type		
	n	%		n	%		n	%		n	%
Female	49	46	1º CSE	12	11	YES	7	7	Team	6	6
									Individual	1	1
						NO	5	5	-	5	
			2º CSE	8	8	YES	7	7	Team	5	5
									Individual	2	2
						NO	1	1	-	1	1
			3º CSE	7	7	YES	5	5	Team	5	5
									Individual	-	-
						NO	2	2	-	2	2
			4º CSE	22	21	YES	18	17	Team	7	7
									Individual	11	10
						NO	4	4	-	4	4
Male	57	54	1º CSE	12	11	YES	11	10	Team	6	6
									Individual	5	5
						NO	1	1	-	1	1
			2º CSE	17	16	YES	16	15	Team	13	12
									Individual	3	3
						NO	1	1	-	1	1
			3º CSE	14	13	YES	12	11	Team	7	7
									Individual	5	5
						NO	2	2	-	2	2
			4º CSE	14	13	YES	13	12	Team	11	10
									Individual	2	2
						NO	1	1	-	1	1
Total	106	100		106	100		106	100		106	100

Note: CSE = Compulsory Secondary Education.

**Table 3:** Pearson’s chi-square (X^2^) among all variables in the study.

	Anxiety	Stress	Depression
Gender	365.65 *	348.02 *	442.55
Educational stages	397.56	477.81 *	493.31
Physical activity	303.34 **	310.64 **	324.32 **
Sport type	172.34 *	179.33 *	188.45

* *p* < 0.05. ** *p* < 0.01.

**Table 4:** Adjusted residuals test, Odds ratio, Confidence Interval and *p*-value: Gender: Psychological well-being.

Gender	Anxiety				Stress				Depression			
	AR	OR	CI 95%	*p*	AR	OR	CI 95%	*p*	AR	OR	CI 95%	*p*
Female	2.23 *	1.82	1.45–2.29	<0.001	2.12 *	1.67	1.32–2.11	<0.001	1.70	2.03	1.61–2.56	<0.001
Male	−2.23 *				−2.12 *				1.70			

Note: AR = Adjusted residuals; OR = Odds Ratio; CI = Confidence Interval; * *p* < 0.05.

**Table 5:** Adjusted residuals test, Odds ratio, Confidence Interval and *p*-value: Educational stages: Psychological well-being.

Educational Stages	Anxiety				Stress				Depression			
	AR	OR	CI 95%	*p*	AR	OR	CI 95%	*p*	AR	OR	CI 95%	*p*
1º/2º CSE	1.05	1.34	1.02–1.76	0.036	2.45 *	1.58	1.21–2.07	<0.001	1.04	1.41	1.09–1.83	0.009
3º/4º CSE	−1.05				−2.45 *				1.04			

Note: CSE = Compulsory Secondary Education; AR = Adjusted residuals; OR = Odds Ratio; CI = Confidence Interval; * *p* < 0.05.

**Table 6:** Adjusted residuals test, Odds ratio, Confidence Interval and *p*-value: Physical activity: Psychological well-being.

Physical Activity	Anxiety				Stress				Depression			
	AR	OR	CI 95%	*p*	AR	OR	CI 95%	*p*	AR	OR	CI 95%	*p*
Athletes	−3.12 **	2.47	1.89–3.23	<0.001	−2.93 **	2.12	1.63–2.76	<0.001	−2.74 **	2.68	2.04–3.53	<0.001
Non-athletes	3.12 **				2.93 **				2.74 **			

Note: AR = Adjusted residuals; OR = Odds Ratio; CI = Confidence Interval; ** *p* < 0.01

**Table 7:** Adjusted residuals test, Odds ratio, Confidence Interval and *p*-value: Sport type: Psychological well-being.

Sport Type	Anxiety				Stress				Depression			
	AR	OR	CI 95%	*p*	AR	OR	CI 95%	*p*	AR	OR	CI 95%	*p*
Individual	2.23 *	1.21	0.95–1.54	0.112	1.97 *	1.29	1.01–1.65	0.043	1.12	1.36	1.06–1.75	0.016
Colective	−2.23				−1.97 *				−1.12			

Note: AR = Adjusted residuals; OR = Odds Ratio; CI = Confidence Interval; * *p* < 0.05.

## Data Availability

The data presented in this study are available on request from the corresponding author due to privacy concerns.

## Abbreviations

The following abbreviations are used in this manuscript:

DASS-21	Depression, Anxiety, and Stress Scale–21
CSE	Compulsory Secondary Education

## References

[1] Laurier C., Pascuzzo K., Jubinville V., Lemieux A. (2024). Physical activity and its benefits on adolescents’ mental health through self-esteem. Front. Child Adolesc. Psychiatry.

[2] Nobre J., Oliveira A.P., Monteiro F., Sequeira C., Ferré-Grau C. (2021). Promotion of mental health literacy in adolescents: A scoping review. Int. J. Environ. Res. Public Health.

[3] Gulliver A., Griffiths K.M., Christensen H. (2010). Perceived barriers and facilitators to mental health help-seeking in young people: A systematic review. BMC Psychiatry.

[4] Morillo-Sarto H., Torres-Vallejos J., Usán P., Barrada J.R., Juarros-Basterretxea J. (2025). The impact of social media disorder, family functioning, and community social disorder on adolescents’ psychological distress: The mediating role of intolerance to uncertainty. Children.

[5] Alkhamees A.A., Alrashed S.A., Alzunaydi A.A., Almohimeed A.S., Aljohani M.S. (2020). The psychological impact of COVID-19 pandemic on the general population of Saudi Arabia. Compr. Psychiatry.

[6] Yue C.L., Ge X., Liu M., Zhang B., Koda S., Yan C. (2022). The association between physical activity and mental health in medical postgraduates in China during COVID-19 pandemic. Front. Psychiatry.

[7] Dahl R., Allen N., Wilbrecht L., Suleiman A. (2018). Importance of investing in adolescence from a developmental science perspective. Nature.

[8] Pfeifer J.H., Allen N.B. (2021). Puberty initiates cascading relationships between neurodevelopmental, social, and internalizing processes across adolescence. Biol. Psychiatry.

[9] Merino A., Berbegal A., Arraiz A., Sabirón F. (2021). Motivación en la adolescencia y el acompañamiento para la autodeterminación. Orientación Soc..

[10] Rickwood D.J., Deane F.P., Wilson C.J. (2007). When and how do young people seek professional help for mental health problems?. Med. J. Aust..

[11] Fazel M., Hoagwood K., Stephan S., Ford T. (2014). Mental health interventions in schools in high-income countries. Lancet Psychiatry.

[12] He J.P., Paksarian D., Merikangas K.R. (2018). Physical activity and mental disorder among adolescents in the United States. J. Adolesc. Health.

[13] Biddle S.J.H., Asare M. (2011). Physical activity and mental health in children and adolescents: A review of reviews. Br. J. Sports Med..

[14] Strong W.B., Malina R.M., Blimkie C.J.R., Daniels S.R., Dishman R.K., Gutin B., Hergenroeder A.C., Must A., Nixon P.A., Pivarnik J.M. (2005). Evidence based physical activity for school-age youth. J. Pediatr..

[15] World Health Organization (2010). Global Recommendations on Physical Activity for Health.

[16] Bover B.M., Arnal R.B., Llario M.D.G., Miravet M.E., Galdón M.L.F. (2020). Motivaciones para el ejercicio físico y su relación con la salud mental y física: Un análisis desde el género. Rev. INFAD Psicología. Int. J. Dev. Educ. Psychol..

[17] Myśliwiec N.V., Ciesielska A., Wojtczak M., Sieradzka A., Kot A., Różycki A., Szerej K. (2025). The impact of physical activity on mental health. Qual. Sport.

[18] Instituto Nacional de Estadística, Ministerio de Sanidad (2023). Encuesta Nacional de Salud En España: Actividad Física en Población Infantil y Adolescente.

[19] World Health Organization (2022). Global Status Report on Physical Activity 2022: Country Profiles.

[20] Santos R.M.S., Mendes C.G., Sen Bressani G.Y., De Alcantara Ventura S., De Almeida Nogueira Y.J., De Miranda D.M., Romano-Silva M.A. (2023). The associations between screen time and mental health in adolescents: A systematic review. BMC Psychol..

[21] Lissak G. (2018). Adverse physiological and psychological effects of screen time on children and adolescents: Literature review and case study. Environ. Res..

[22] Chahín-Pinzón N., Briñez B. (2011). Actividad física en adolescentes y su relación con agresividad, impulsividad, internet y videojuegos. Psychol. Av. Discip..

[23] Biernat E., Piątkowska M., Rozpara M. (2022). Is the prevalence of low physical activity among teachers associated with depression, anxiety, and stress?. Int. J. Environ. Res. Public Health.

[24] Gasol Foundation (2021). Estudio PASOS 2019: Evaluación del Nivel de Actividad Física, Sedentarismo y Obesidad en Población Infantil y Adolescente en España.

[25] Pate R.R., Davis M.G., Robinson T.N., Stone E.J., McKenzie T.L., Young J.C. (2006). Promoting physical activity in children and youth: A leadership role for schools. Circulation.

[26] Fernández-Sogorb A., Jiménez-Ayala C.E., Cargua N.I., Aparicio-Flores M.P., Antón N., García-Fernández J.M. (2024). Does school climate affect student well-being? Anxiety in school situations as a predictor of stress in high-school students. J. Educ. Health Promot..

[27] Bisquerra Alzina R. (2003). Educación emocional y competencias básicas para la vida. Rev. Investig. Educ..

[28] Silva L.A., Doyenart R., Salvan P.H., Rodrigues W., Lopes J.F., Gomes K., Thirupathi A., Pinho R.A.D., Silveira P.C. (2020). Swimming training improves mental health parameters, cognition and motor coordination in children with ADHD. Int. J. Environ. Health Res..

[29] Ishihara T., Sugasawa S., Matsuda Y., Mizuno M. (2017). Improved executive functions in 6–12-year-old children following cognitively engaging tennis lessons. J. Sports Sci..

[30] Kajtna T., Vuleta D., Pori M., Justin I., Pori P. (2012). Psychological characteristics of Slovene handball goalkeepers. Kinesiology.

[31] Madinabeitia-Cabrera I., Alarcón-López F., Chirosa-Ríos L.J., Pelayo-Tejo I., Cárdenas-Vélez D. (2023). The cognitive benefits of basketball training compared to combined endurance and resistance training: A four-month intervention study. Sci. Rep..

[32] Guo C., Chen F., Wang Y. (2025). The psychological impact of competitive sports participation on adolescent athletes: An analysis of coping mechanisms and performance outcomes. J. Rat.-Emot. Cogn.-Behav. Ther..

[33] Xue Y., Yang Y., Huang T. (2019). Effects of chronic exercise interventions on executive function among children and adolescents: A systematic review with meta-analysis. Br. J. Sports Med..

[34] Yang C., Luo N., Liang M., Zhou S., Yu Q., Zhang J., Zhang M., Guo J., Wang H., Yu J. (2020). Altered brain functional connectivity density in fast-ball sports athletes with early stage of motor training. Front. Psychol..

[35] Voss M.W., Nagamatsu L.S., Liu-Ambrose T., Kramer A.F. (2011). Exercise, brain, and cognition across the life span. J. Appl. Physiol..

[36] Wu Q., Tan Y., Sun G., Ding Q. (2023). The relationship between self-concept clarity, athletic identity, athlete engagement and the mediating roles of quality of life and smartphone use in Chinese youth athletes. Heliyon.

[37] Berki T., Tarjányi Z. (2022). The role of physical activity, enjoyment of physical activity, and school performance in learning motivation among high school students in Hungary. Children.

[38] Wang Q., Zainal Abidin N.E., Aman M.S., Wang N., Ma L., Liu P. (2024). Cultural moderation in sports impact: Exploring effects on educational progress, cognitive focus, and social development. BMC Psychol..

[39] Zarazaga-Peláez J., Barrachina V., Gutiérrez-Logroño A., Villanueva-Guerrero O., Roso-Moliner A., Mainer-Pardos E. (2024). Impact of Extracurricular Physical Activity on Achievement of the Sustainable Development Goals and Academic Performance: Mediating Cognitive, Psychological, and Social Factors. Sustainability.

[40] Vaughan R.S., Edwards E.J., MacIntyre T.E. (2020). Mental health measurement in a post Covid-19 world: Psychometric properties and invariance of the DASS-21 in athletes and non-athletes. Front. Psychol..

[41] Rodriguez-Ayllon M., Cadenas-Sánchez C., Estévez-López F., Muñoz N.E., Mora-Gonzalez J., Migueles J.H., Molina-García P., Henriksson H., Mena-Molina A., Martínez-Vizcaíno V. (2019). Role of physical activity and sedentary behavior in the mental health of youth: A systematic review and meta-analysis. Sports Med..

[42] Guthold R., Stevens G.A., Riley L.M., Bull F.C. (2020). Global trends in insufficient physical activity among adolescents: A pooled analysis of 298 surveys. Lancet Child Adolesc. Health.

[43] Eime R.M., Young J.A., Harvey J.T., Charity M.J., Payne W.R. (2013). A systematic review of the psychological and social benefits of participation in sport. Int. J. Behav. Nutr. Phys. Act..

[44] Chen L., Liu Q., Xu F., Wang F., Luo S., An X., Chen J., Tang N., Jiang X., Liang X. (2024). Effect of physical activity on anxiety, depression and obesity in children and adolescents with obesity: A meta-analysis. J. Affect. Disord..

[45] Wang X., Cai Z., Jiang W., Fang Y., Sun W., Wang X. (2022). Systematic review and meta-analysis of the effects of exercise on depression in adolescents. Child Adolesc. Psychiatry Ment. Health.

[46] Lubans D., Richards J., Hillman C., Faulkner G., Beauchamp M., Nilsson M., Kelly P., Smith J., Raine L., Biddle S. (2016). Physical activity for cognitive and mental health in youth: A systematic review of mechanisms. Pediatrics.

[47] Bailey R., Hillman C., Arent S., Petitpas A. (2013). Physical activity: An underestimated investment in human capital?. J. Phys. Act. Health.

[48] Nolen-Hoeksema S. (2012). Emotion regulation and psychopathology: The role of gender. Annu. Rev. Clin. Psychol..

[49] Li S.H., Graham B.M. (2017). Why are women so vulnerable to anxiety, trauma-related and stress-related disorders? The potential role of gender hormones. Lancet Psychiatry.

[50] Schrijvers D.L., Bollen J., Sabbe B.G.C. (2012). The gender paradox in suicidal behavior and its impact on the suicidal process. J. Affect. Disord..

[51] Compas B.E., Jaser S.S., Bettis A.H., Watson K.H., Gruhn M.A., Dunbar J.P., Williams E., Thigpen J.C. (2017). Coping, emotion regulation, and psychopathology in childhood and adolescence: A meta-analysis. Psychol. Bull..

[52] Reddy K.J., Menon K.R., Thattil A. (2018). Academic stress and its sources among university students. Biomed. Pharmacol. J..

[53] Patton G.C., Sawyer S.M., Santelli J.S., Ross D.A., Afifi R., Allen N.B., Arora M., Azzopardi P., Baldwin W., Bonell C. (2016). Our future: A Lancet commission on adolescent health and wellbeing. Lancet.

[54] Orben A., Tomova L., Blakemore S.J. (2020). The effects of social deprivation on adolescent development and mental health. Lancet Child Adolesc. Health.

[55] Biddle S., Ciaccioni S., Thomas G., Vergeer I. (2019). Physical activity and mental health in children and adolescents: An updated review of reviews and analysis of causality. Psychol. Sport. Exerc..

[56] Pluhar E., McCracken C., Griffith K.L., Christino M.A., Sugimoto D., Meehan W.P. (2019). Team sport athletes may be less likely to suffer anxiety or depression than individual sport athletes. J. Sports Sci. Med..

